# Groin surgical site infection incidence in vascular surgery with intradermal suture versus metallic stapling skin closure: A study protocol for a pragmatic open-label parallel-group randomized clinical trial (VASC-INF trial)

**DOI:** 10.1097/MD.0000000000031800

**Published:** 2022-12-16

**Authors:** Albert González-Sagredo, Miquel Gil, Mario D’Oria, Konstantinos Spanos, Álvaro Salinas, Selene Matus, Thiago Carnaval, Secundino Llagostera, Sandro Lepidi, Athanasios Giannoukas, Sergi Bellmunt, Raul García-Vidal, Sebastián Videla, Ramon Vila, Elena Iborra

**Affiliations:** a Angiology and Vascular Surgery Department, Bellvitge University Hospital, L’Hospitalet de Llobregat, Barcelona, Spain; b Angiology and Vascular Surgery Department, Hospital Germans Tries i Pujol, Badalona, Barcelona, Spain; c Division of Vascular and Endovascular Surgery, University Hospital of Trieste ASUGI, Trieste, Italy; d Angiology and Vascular Surgery Department, Larissa University Hospital, Larissa, Greece; e Angiology and Vascular Surgery Department, Vall d’Hebrón University Hospital, Barcelona, Spain; f Angiology and Vascular Surgery Department, Joan XXIII University Hospital, Tarragona, Spain; g Clinical Research Support Unit, Clinical Pharmacology Department, Bellvitge University Hospital, L’Hospitalet de Llobregat, Barcelona, Spain; h Pharmacology Unit, Department of Pathology and Experimental Therapeutics, School of Medicine and Health Sciences, IDIBELL, University of Barcelona, L’Hospitalet DE Llobregat, Barcelona, Spain.

**Keywords:** intradermal suture, metallic stapling, surgical site infection

## Abstract

Surgical site infection is 1 of the most frightening complications in vascular surgery due to its high morbimortality. The use of intradermal sutures for skin closure might be associated with a reduction in infections incidence. However, the data available in the literature is scarce and primarily built on low-evidence studies. To our knowledge, no multicenter clinical trial has been published to assess if the intradermal suture is associated with a lower surgical site infection incidence than metallic staples in patients who will undergo revascularization surgery requiring a femoral approach.

**Methods:**

VASC-INF is a pragmatic, multicenter, multistate (Spain, Italy, and Greece), randomized, open-label, clinical trial assessing the surgical site infection incidence in patients undergoing revascularization surgery requiring a femoral approach. Patients will be randomized on a 1:1 ratio to intradermal suture closure (experimental group) or to metallic staples closure (control group).

The primary outcome is the number (percentage) of patients with surgical site infection (superficial and/or deep) associated with a femoral approach up to 28 (±2) days after surgery. Among the secondary outcomes are the number (percentage) of patients with other surgical wound complications; the number (percentage) of patients with surgical site infections who develop sepsis; type of antibiotic therapy used; type of microorganisms’ species isolated and to describe the surgical site infection risk factors.

**Discussion:**

Intradermal suture closure may be beneficial in patients undergoing revascularization surgery requiring a femoral approach. Our working hypothesis is that intradermal suture closure reduces the incidence of surgical site infection respect to metallic staples closure.

## 1. Introduction

### 1.1. Background and rationale

Surgical site infection (SSI) is an infection that occurs after surgery in the part of the body where the surgery took place.^[1,2]^ They are among the most common and fearsome complications in vascular surgery, given their high morbidity and mortality rates,^[3]^ and can be either superficial or deep, depending on their characteristics.^[4]^

SSI are associated with a 2 to 3-fold increased risk of death and a 60% increased risk of requiring postoperative intensive care support. They increase the length of hospital stay by 7 to 12 days; patients are 5 times more likely to require readmission, and direct healthcare costs raise at least US$5000.^[5–7]^ The groin is the most common infection site in vascular surgery since it is a folding area full of lymph glands and located close to genitalia.^[8]^ SSI incidence after vascular surgery has dropped from almost 30%^[9]^ in the 1980s to 20%^[10]^ in the 2000s and currently is approximately 10%,^[11]^ which may be due to improvements in asepsis measures and operative time.

A 2021 meta-analysis on groin SSI prevention strategies found lower rates of SSI associated with intradermal sutures.^[12]^ In a retrospective patient record study of 330 groin incisions (performed in 256 patients), 262 patients were closed with intradermal suture and 9.2% presented SSI. The remaining 68 incisions were closed with metallic stapling, from which 25% presented SSI.^[13]^ Likewise, a retrospective study comparing the groin SSI rate associated with the inguinal closure technique (i.e., intradermal suture vs metallic staples), showed that 89% of SSIs were related to staples, and only 11% to intradermal sutures.^[14]^

We designed a pragmatic, multinational (Spain, Italy, and Greece), randomized, open-label, clinical trial (VASC-INF) to assess the SSI incidence in patients undergoing revascularization surgery requiring a femoral approach. We aim to demonstrate the superiority of skin closure with intradermal sutures (experimental group) compared to metallic stapling (control group) in terms of reducing the incidence of SSI (superficial and/or deep) associated with arterial vascular intervention through a groin incision.

### 1.2. Objectives

Our primary objective is to estimate the SSI incidence (superficial and/or deep) associated with a femoral approach up to 28 (±2) days after surgery.

Our secondary objectives are as follows:

To estimate the incidence of other surgical wound complications (e.g., seroma, hematoma, lymphorrhagia) associated with a femoral approach up to 28 (±2) days after surgery.
To estimate the incidence of sepsis in patients with femoral approach-related SSI up to 28 (±2) days after surgery.
To describe the timing of prophylactic antibiotic administration.
To describe the microorganisms’ species isolated on the microbiological culture of skin, subcutaneous tissue, and/or SSI secretion sample up to 28 (±2) days after surgery.
To describe the antibiotic therapy used in patients with femoral approach-related SSI up to 28 (±2) days after surgery.
To assess the association between nutritional status (measured by serum albumin levels) and femoral approach-related SSI up to 28 (±2) days after surgery.
To assess the association between body mass index (BMI) and femoral approach-related SSI up to 28 (±2) days after surgery.
To assess the association between incision length and femoral approach-related SSI up to 28 (±2) days after surgery.
To assess the association between surgery duration and femoral approach-related SSI up to 28 (±2) days after surgery.
To assess the association between the kind of surgery performed and femoral approach-related SSI up to 28 (±2) days after surgery.
To assess the association between length of hospital stay before surgery and femoral approach-related SSI up to 28 (±2) days after surgery.
To assess the association between foot lesions (Rutherford ischemic stages 5 or 6) and femoral approach-related SSI up to 28 (±2) days after surgery.
To estimate the SSI incidence (superficial and/or deep) associated with the femoral approach up to 84 (±7) days after surgery.

### 1.3. Trial design

This is a study protocol of an open-label, pragmatic, multinational, randomized clinical trial (VASC-INF). All adult patients undergoing revascularization surgery requiring a femoral approach who meet all the inclusion criteria and none of the exclusion criteria will be enrolled in this trial. Patients will be informed about the trial and will be invited to participate. All potential participants will be notified that the surgical procedure will be conducted per standard clinical practice. We will only randomize the type of skin closure (i.e., intradermal sutures or metallic staples).

## 2. Materials and methods

### 2.1. Study setting

VASC-INF will be performed in 3 countries: Spain, Italy, and Greece, and the following tertiary hospitals will participate: Bellvitge University Hospital, Germans Trias i Pujol University Hospital, Joan XXIII University Hospital, Vall d’Hebron University Hospital, Trieste University Hospital, and Larissa University Hospital. These are all specialized healthcare centers with all the means necessary to carry out complex surgeries.

### 2.2. Eligibility criteria

Patients can be enrolled in the trial if all of the following criteria apply:

Adult patients (≥18 years old).
Both genders.
Diagnosed with chronic lower limb ischemia or aortic, iliac, or femoral aneurysm.
With a scheduled surgery for 1 of the following indications:
  Femoropopliteal bypass.
  Femorodistal bypass.
  Aortobifemoral bypass.
  Axillofemoral or axillobifemoral bypass.
  Femorofemoral bypass.
  Femoral endarterectomy.
  Femoral approach for exclusion of an aortic aneurysm.
Surgical procedure with an incision perpendicular to the groin fold.
Patients who undergo both unilateral or bilateral surgical approaches.
  **Note**: we will consider 1 patient as 1 intervention (i.e., bilateral approaches will be quantified as 1 single femoral approach). In the case of bilateral procedures, the closure technique will be the same for both sides.
Patients who sign the written informed consent.

Patients will be automatically excluded if any of the following criteria apply:

Background of a previous surgical intervention in the groin.
Femoral approach carried out in a surgical emergency setting.
Femoral approach performed due to a femoral pseudoaneurysm.
A surgical procedure performed with a transverse/oblique incision to the inguinal fold.
A patient who withdraws consent to participate in the trial.

### 2.3. Interventions

Description.

Once the subjects or their legally authorized representative sign the written informed consent, the study will begin. Randomization will be performed by a study team member on the day of the surgery, and only the circulating nurse will be aware of the result. Antibiotic prophylaxis will be administered and registered. Groin area hair clipping will be performed before entering the operating room. Then, asepsis of the surgical area will be served with 4% chlorhexidine and a surgical drape will be placed. The surgical intervention will be performed with an incision perpendicular to the inguinal fold. For groin closure, the fascia and the subcutaneous tissue will be closed with a continuous absorbable filament suture (Vycril [Novosyn® 2/0]). At this moment, the circulating nurse will inform about the closure technique (as determined by random assignment). Both skin closure techniques are described as follows:

Intradermal suture (Monosyn Braun® 4/0 absorbable monofilament): the surgeon chooses 1 of the wound’s apexes to settle an anchoring dermal stitch. Subsequently, performing a mirror image at the opposing side, placing a dermal stitch at 0.5 centimeters from the wound edge and always trying to keep the bite at the same length. The surgeon then alternates the stitching sides until reaching the other apex of the wound, performing 5 knots to secure the suture. Afterwards, passes the needle back deep through the edge, emerging it adjacent to the wound, thereby burring the knot.
Metallic stapling: the surgeon everts 1 of the edges of the skin. Likewise, the assistant everts the other edge. The surgeon then staples both skin edges together by applying pressure and activating the stapler mechanism. The staples should be placed 0.5 centimeters apart and cover the entire incision length.

Once the skin closure has been performed, a surgical dressing will be placed on the groin area, thereby finishing the surgery. The surgical dressing will not be uncovered for a 48-hours timeframe unless strictly necessary.

Criteria for discontinuing or modifying allocated interventions.

Given the characteristics of this clinical trial, once the randomly assigned skin closure procedure has been performed, it cannot be modified. Participants may voluntarily discontinue their participation in the clinical trial for any reason, at any time. The investigator may also decide at any time during the trial to temporarily interrupt or permanently discontinue the patients’ participation in the clinical trial if it is deemed that continuation would be detrimental to or not in the participant’s best interest. Similarly, the ethics committee or authorized regulatory authority can decide to halt or prematurely terminate the trial when new information becomes available whereby the rights, safety and well-being of trial participants can no longer be assured, when the integrity of the trial has been compromised, or when the scientific value of the trial has become obsolete and/or unjustifiable.

Strategies to improve adherence to intervention protocols.

Once the surgical wound has been closed, the study intervention will no longer be changeable.

Relevant concomitant care permitted or prohibited during the trial.

No concomitant care is prohibited during the trial and postoperative care will be performed according to local standard clinical practice guidelines.

### 2.4. Outcomes

Our primary outcome measure is the number (percentage) of patients who present a femoral approach SSI up to 28 (±2) days after surgery.

Our secondary outcome measures are:

Number (percentage) of patients with other surgical wound complications (e.g., seroma, hematoma, lymphorrhagia) associated with a femoral approach up to 28 (±2) days after surgery.
Number (percentage) of patients with femoral approach SSI who develop sepsis up to 28 (±2) days after surgery.
Time of prophylactic antibiotic administration.
Type of microorganisms’ species isolated on the microbiological culture of skin, subcutaneous tissue, and/or SSI secretion sample up to 28 (±2) days after surgery.
Type of antibiotic therapy used in patients with femoral approach-related SSI up to 28 (±2) days after surgery).
Serum albumin levels.
BMI.
Surgery duration.
Number of days between hospital admission and surgical intervention.
Number (percentage) of patients who present a femoral approach SSI up to 84 (±7) days after surgery.
Kind of surgery performed.
Presence of foot lesions (Rutherford ischemic stages 5 or 6).

### 2.5. Participant timeline

Participant timeline is summarized in Table 1. The entire clinical trial is expected to last approximately 2.5 years. The first patient will be included in April 2022, and the last patient is expected to be included in October 2023. Afterwards, trial publication is scheduled for October 2024.

**Table 1:** Participant timeline. Surgical site infection (SSI).

	Baseline visit Screening	Visit 1 Surgery	Visit 2 Hospitalization	Visit 3 28 (±2) d after surgery	Visit 4 84 (±7) d after surgery
Eligibility criteria	X				
Written informed consent	X				
Demographic data	X				
Medical history	X				
Medical examination	X	X	X	X	
Surgical intervention data		X			
SSI register			X	X	X
Hospital readmission register				X	X

SSI = surgical site infection.

### 2.6. Schedule of assessments

Baseline visit (Screening and Enrollment): the baseline visit will be primarily performed in the Outpatient Clinic. However, if the subject is an inpatient, it will be conducted during hospitalization. A clinical evaluation will assess if the subject meets all the inclusion criteria and none of the exclusion criteria. The subject will be informed about the trial procedures, as well as about their risks and benefits. The Patient Information Sheet (containing complete trial procedures’ information) will be handled, and the subject or their legally authorized representative will be asked to sign the written informed consent. Once the written informed consent has been signed, the subject automatically becomes an enrolled study participant. Subsequently, we will gather demographic data, BMI, nutritional assessment (i.e., serum albumin levels), past medical history and medical examination data. As in standard clinical practice, we will make preoperative assessments (chest radiography, electrocardiogram, and blood analysis).
Visit 1 (Day of the Surgical Intervention): participants will be randomly assigned to 1 of the study groups right before surgery through the electronic case report form (eCRF) itself (research electronic data capture software [REDCap®] platform). After surgery, the study team will record several data: the type of anesthesia, any incident during the surgical intervention, the procedures’ duration, surgical wound length, the material used (vein, dacron, polytetrafluoroethylene, omniflow), closure technique performed and degree of the surgeon performing the incision and the closure (a trainee or a staff member).
Visit 2 (Hospital Stay): the surgical dressing will be uncovered 48 hours after surgery. Patients assigned to the control group (i.e., metallic stapling skin closure) will have their staples removed 7/10 days after surgery. Patients discharged before the 7^th^ day will have their staples removed in the Outpatient Clinic. During the hospital stay, we will perform a daily surgical wound inspection. Postoperative blood tests and complications will be recorded. This data will be entered into the program on the day of hospital discharge.
Visit 3 (at 28 [±2] days after surgery): a face-to-face visit will be scheduled. The investigator will record (if any) the number and reason for extra medical consultation (including Emergency Department, hospital readmissions, and primary care either face-to-face or by phone). We will perform a surgical wound exam looking for any sign of SSI or other possible local complications (e.g., seroma, hematoma, lymphorrhagia). We will record the microbiological culture results that could be pending by the time of hospital discharge.
Visit 5 (at 84 [±7] days after surgery): It will be a phone interview. The investigator will record (if any) the number and reason for extra medical visits (including Emergency Department, hospital readmissions, and primary care either face-to-face or by phone). We will ask about any symptoms related to surgical wound complications, and in case of detecting any alarming signs a face-to-face visit will be scheduled. The participant will be informed about the end of the study period and perform a standard follow-up protocol.

### 2.7. Sample size

Our primary endpoint is the number (percentage) of patients who present a femoral approach SSI up to 28 (±2) days after surgery. We intend to contrast:

Null hypothesis: there is no difference in the SSI incidence related to the femoral approach between the metallic stapling skin closure and the intradermal suture skin closure.
Alternative hypothesis: the SSI incidence related to the femoral approach when performing a metallic stapling closure differs from the incidence when performing an intradermal suture skin closure.

To our knowledge, no published prospective studies compare SSI incidence between staples and intradermal closure. Nonetheless, a 2018 retrospective Finnish study estimated an SSI incidence of 9.2% for intradermal suture skin closure and 25.0% for metallic stapling skin closure.^[13]^ Under this assumption, 200 patients (100 per study group) would be necessary to reject the null hypothesis at a 5% significance level and a power of 80% using a 2-sided χ ^[2]^ test with a 1:1 allocation to treatment groups. Accounting a possible dropout rate of 10%, 224 subjects should be enrolled (112 per study group).

### 2.8. Recruitment

Each hospital involved will screen subjects until the target population of 224 patients is achieved. Given that patients will be recruited following standard clinical practice procedures, the recruited study population should provide generalizable results.

### 2.9. Allocation, sequence generation, concealment, implementation, and blinding

Participants will be randomly assigned into 2 treatment groups: the control group (metallic stapling) or the experimental group (intradermal sutures). The randomization process will be performed by a study member on the day of the surgery and will be centralized electronically using REDCap-based eCRF. The randomization list will be computer-generation in a 1:1 random size block distribution. The surgical team will not know the result of the randomization until the closure of the subcutaneous tissue is started. At that moment, the circulating nurse will inform the randomized closure technique.

Since this is an open-label study, no concealment mechanism or blinding (masking) technique will be applied.

### 2.10. Data collection and management

Data collection methods:

The study team will gather all study-related data through an *ad hoc*-created database. We will collect data on enrollment date, demographic and medical patients’ data (age, gender, BMI, smoking history, past medical history [hypertension, diabetes mellitus, dyslipidemia, ischemic cardiomyopathy, stroke, chronic obstructive pulmonary disease, chronic kidney disease defined as a glomerular filtration rate ≤60mL/min]), laboratory results (plasma albumin levels, white blood cells, hemoglobin, platelets, sodium, potassium, creatinine, glomerular filtration rate), medical exam, surgical indication (and in case of Rutherford ischemic stages 5 or 6, if the ulcers are infected), surgical procedure data, postoperative complications, and hospital readmission record (number and reason).

Data collection will be carried out by the collaborating researcher using an *ad hoc*-created eCFR. Clinical and analytical data described in the study outcome measures will be collected from medical history during admission and scheduled follow-up. The REDCap® Platform automatically generates an anonymized database with all the information provided through the eCRF. The same study group will manage this database. Enrolled participants will be assigned a number to ensure anonymity. All gathered data will be stored in a secure and accessible way. The records will be retained for 3 years after completing the study. The investigator will keep all documents and data relating to the study in a secure file and/or electronically. The storage system used during the research and for archiving (irrespective of the media used) should provide document identification, version history, search, and retrieval. The data will be available for evaluation and/or audits from the study team and Health Authorities. The appropriate measures regarding data confidentiality will be adopted according to the Spanish Organic Law 3/2018 on the Protection of Personal Data. During the study, data monitoring will be performed to review the information gathered for security reasons, verify protocol adherence, and contrast the database information with the source document (which should ensure the quality of generated data).

Participant retention.

During follow-up, the investigator will remind the patient of the importance of correctly following the study procedures and encourage them to continue in the clinical trial. Protocol deviations will be documented and explained in detail by the study team. If a “serious” protocol violation occurs, the monitoring team will record all protocol breaches/deviations. According to international conference on harmonization (ICH) Good Clinical Practice Guidelines, the Sponsor will review all protocol deviations and assess whether any of them represent a “serious” violation according to ICH. The Sponsor will inform the institutional review board (IRB) of any protocol breach/deviation that could impact patient safety and/or data integrity.

### 2.11. Statistical methods

All analyses related to the intervention efficacy will be based on the intention-to-treat (ITT) population, keeping all patients selected and randomized in the group in which they were initially included, regardless of adherence to the protocol. Safety analyses will be based on the safety population who have been operated on after randomization. The demographic and clinical profiles of all included subjects will be described by the study group using statistics according to variable type.

The primary outcome measure will be compared using the ^[2]^ test. The magnitude of the intervention effect will be presented as a cumulative incidence ratio at 28 (±2) days after surgery, along with a 95% confidence interval (95%CI). In the secondary outcomes, incidences will be compared using the ^[2]^ test and the magnitude of the effect will be presented as a cumulative incidence ratio along with a 95%CI. A regression logistic model will be used to assess the effect of nutritional status, BMI, incision length, surgery duration, type of surgery, length of hospital before surgery, and femoral approach-related SSI up to 28 (±2) days after surgery.

Analysis will be performed using R® version 4.1.0 for Windows® (R Core Team [2020]. R: A language and environment for statistical computing. R Foundation for Statistical Computing, Vienna, Austria. URL: https://www.Rproject.org/).

### 2.12. Interim analyses

An interim analysis will be performed 9 months after the beginning of the trial, when we expect to have enrolled approximately half of the estimated sample size.

### 2.13. Handling protocol non-adherence and missing data

In the case of missing data, imputation will be made using the Last Observation Carried Forward.

### 2.14. Data monitoring, harms, and auditing

The trial steering committee will be made up by AGS, EI and SV. The investigators will seek adverse events (AE) at every scheduled follow-up visit. Any adverse event, whether related or not to the study, will be recorded in the eCRF and coded using the latest version of MedDRA (Medical Dictionary for Regulatory Activities). The investigator will also provide severity and causality assessments when fulfilling the AE section of the eCRF. AEs will be described using absolute and relative frequencies by study group, according to the severity and causal relation with the experimental and control interventions. Likewise, severe AEs will be described for each study group, and the 95%CI of the difference between groups will be calculated. The investigator shall allow direct access to trial data and documents for monitoring, audits, and inspections by the competent regulatory or healthcare authorities. As such, eCRFs, source records, and other trial-related documents must be kept up-to-date, complete, and accurate at all times.

### 2.15. Ethics and dissemination

Research ethics approval.

This trial will be conducted according to the criteria set by the declaration of Helsinky, ICH good clinical practice standards, and applicable regulations. Patients will be informed that their participation in the trial is entirely voluntary and that they can withdraw their consent at any time, under no penalty risk whatsoever. The Investigator’s participation in this study is free, voluntary, unpaid, and independent. By the current legislation (Royal Decree 1090/2015), this clinical trial has a low level of intervention since both skin closure techniques are currently used in standard clinical care. The present study protocol was approved by the Bellvitge University Hospital Research Ethics Committee (PR047/22).

Protocol amendments.

Significant protocol changes will be submitted for IBR, and competent regulatory authorities’ approval, and minor outcomes will be informed to the IRB. As per good clinical practice, trial participants will be notified of any significant changes during the trial.

Consent of assent.

The investigators will inform the screened subjects about the study and ask them to sign the written informed consent.

Confidentiality.

The results from this clinical trial are confidential and may not be transferred to 3^rd^ parties in any form or manner without written permission from the Sponsor. All individuals involved in the clinical trial are bound to this confidentiality clause in line with the Regulation (EU) 2016/678 of the European Parliament and of the Council of April 27^th^, 2016, on the protection of natural persons about the processing of personal data and on the free movement of such data, as well as other valid and applicable laws and regulations, such as the Spanish Organic Law 3/2018, of December 5^th^, on Personal Data Protection and Digital Rights Assurance. When obtaining a signature for the Written Informed Consent, the investigator will request written permission from the patient to access his/her data directly. With this permission granted, the patient’s data may be examined, analyzed, verified, and reproduced for the clinical trial evaluation.

Data will be anonymized and dissociated so that the corresponding patient cannot be identified. Patients will be assigned consecutive numbers as they are enrolled in the study, and these identification numbers (or codes) will be used in the eCRF; the full name of the patient will not be included in the eCRFs. The principal investigator of each center will keep an updated patient identification list containing the name, clinical history number, and the patient’s identification number (or code) for the clinical trial. The study monitor may access the patient’s identity and data related to the study monitoring procedures. Anyone with direct access to the data (Regulatory Authorities, Trial Monitors, and auditors) will take all possible precautions to maintain the confidentiality of patients’ identities. It is the investigator’s responsibility to obtain written informed consent from the study patients. The Trial Monitor’s responsibility is to ensure that each patient has given their written consent to allow this direct access. The investigator shall ensure that the documents provided to the Sponsor do not contain the patient’s name or any identifiable data.

Conflict of interest/competing interests

All authors have no conflicts of interest to disclose.

Access to data

No public access to participants’ datasets is planned to be given at this moment. The Sponsor will oversee the dataset. Granting access to this information will be evaluated on a case-by-case basis and upon reasonable request by the interested party.

Ancillary and post-trial care

This is a low-level interventional trial since both skin closure techniques are currently used in standard clinical care. The complementary diagnostic of follow-up procedures entails the same safety risks as those conducted in standard clinical practice. Therefore, *ad-hoc* insurance has not been hired for this trial, given that the participants are already covered by individual or collective professional civil liability insurance or equivalent financial guarantee of the healthcare center where the clinical trial will be conducted. Likewise, post-trial care will be performed by the patient’s designated physician, as per standard clinical practice, following the same follow-up protocol that applies to the general population (i.e., that does not participate in the trial).

Dissemination policy

The study finding will be submitted to a peer-reviewed journal for publication and presented at national and international scientific meetings. The authorship will be based on the International Committee of Medical Journal Editors guidelines.

Informed consent and patient information sheet

The informed consent form and the patient information sheet are available in the supplementary materials.

Biological specimens

Suppose any future study is planned to be carried out lateral using the anonymized stored data. In that case, a new protocol should be made, and a new IRB approval should be sought. Currently, the study team does not intend to carry out any ancillary studies.

## 3. Discussion

SSI is 1 of the most frightening complications in vascular surgery.^[3,5–7]^ The rational for this trial is that SSI incidence might be related to the type of skin closure technique performed. However, the evidence is scarce, primarily built on low-evidence studies, and the chosen skin closure technique is mostly decided based on surgeon’s preference.^[11,13,14]^ Therefore, we have designed this RCT to assess if intradermal suture is associated with a lower SSI incidence than staples. We have focused on the arterial femoral approach as the groin is the most prevalent area of infections and is a common approach in vascular surgery.^[8]^ We believe our estimated sample size will represent the overall population, given that the enrolled patients will be extracted from our standard clinical practice. Participants will perceive the same benefits from the surgical procedure as any other patient, without added risks. We expect the results from this trial could help elucidate which skin closure technique associates with a lesser SSI incidence, therefore reducing its related morbidity and mortality.

## Acknowledgements

We would like to thank Cristian Tebé, Pau Satorra and Aurema Otero from the statistical and research department for their collaboration with the development of this protocol.

We also would like to thank the IDIBELL Foundation and the CERCA Program/Generalitat de Catalunya for the institutional support provided.

## Author contributions

**Conceptualization:** Albert González-Sagredo, Miquel Gil, Selene Matus, Sandro Lepidi, Sergi Bellmunt, Sebastián Videla, Ramon Vila, Elena Iborra.

**Data curation:** Albert González-Sagredo, Sebastián Videla, Elena Iborra.

**Formal analysis:** Albert González-Sagredo, Thiago Carnaval, Sebastián Videla.

**Funding acquisition:** Albert González-Sagredo.

**Investigation:** Albert González-Sagredo, Miquel Gil, Thiago Carnaval, Sebastián Videla, Ramon Vila, Elena Iborra.

**Methodology:** Albert González-Sagredo, Miquel Gil, Mario D’Oria, Konstantinos Spanos, Thiago Carnaval, Secundino Llagostera, Athanasios Giannoukas, Sebastián Videla, Ramon Vila, Elena Iborra.

**Project administration:** Albert González-Sagredo.

**Resources:** Secundino Llagostera, Sandro Lepidi.

**Supervision:** Athanasios Giannoukas, Sergi Bellmunt, Raul García-Vidal, Sebastián Videla, Ramon Vila, Elena Iborra.

**Validation:** Albert González-Sagredo, Mario D’Oria, Konstantinos Spanos, Selene Matus, Athanasios Giannoukas, Sergi Bellmunt, Sebastián Videla, Ramon Vila, Elena Iborra.

**Visualization:** Albert González-Sagredo, Álvaro Salinas, Sebastián Videla, Ramon Vila, Elena Iborra.

**Writing – original draft:** Albert González-Sagredo, Thiago Carnaval, Sebastián Videla, Ramon Vila, Elena Iborra.

**Writing – review & editing:** Albert González-Sagredo, Miquel Gil, Mario D’Oria, Konstantinos Spanos, Álvaro Salinas, Selene Matus, Thiago Carnaval, Secundino Llagostera, Sandro Lepidi, Athanasios Giannoukas, Sergi Bellmunt, Raul García-Vidal, Sebastián Videla, Ramon Vila, Elena Iborra.
